# The Potential Anaerobic Cultivation of Microbial Biomass for Astronaut Nutrition During Long-Term Space Flight

**DOI:** 10.3390/life16091469

**Published:** 2026-09-02

**Authors:** Hillary H. Smith, Gregory M. Wong, Sydney N. Assalita, Zhidan Zhang, Christopher H. House

**Affiliations:** Department of Geosciences, The Pennsylvania State University, 220 Deike Building, State College, PA 16802, USAassalita@umich.edu (S.N.A.);

**Keywords:** anaerobic growth, lipids, proteins, single-cell protein, fermentation, controlled ecological life-support system (CELSS)

## Abstract

Long-term crewed space missions will have significant constraints on the weight and volume of supplies. The food needed and the management of the waste produced present a challenge, demanding technologies that effectively recycle nutrients. Using microbiological processes may be a way to meet this challenge. Here, we suggest that byproducts of anaerobic wastewater digestion (organic acids, H_2_, and CO_2_) could be paired with waste CO from atmospheric regeneration to produce microbial biomass, specifically *Acetobacterium woodii* and *Rubrivivax gelatinosus*, as a food source. First, we showed that the thermodynamics of key reactions are favorable. Next, we conducted nutritional analyses to evaluate suitability of *A. woodii* and *R. gelatinosus* as potential food supplements. *A. woodii* biomass consisted of 21% protein and 11% lipid on a dry mass basis. *R. gelatinosus* biomass consisted of 54% protein and 21% lipid on a dry mass basis. Finally, we conducted growth experiments for *R. gelatinosus* under varied (ammonium) acetate concentrations. Following the observation that an intermediate amount of acetate produced the most biomass, a second growth experiment showed that a high level of acetate, rather than ammonium, wasthe observed inhibiting factor. This perspective hopefully inspires new research establishing systems where waste products are recycled via microbial growth during long-term space flight.

## 1. Introduction

Long-duration human space flight requires approaches for astronaut survival using self-contained systems that minimize food supplies and recycle waste produced [1]. The need to reduce waste led to the International Space Station (ISS) water management and recovery system, where liquid astronaut waste is processed into potable water [2,3]. While this advanced technology results in 98% water recovery, nutrients from ISS solid waste are not recycled and instead are either returned to Earth or incinerated via reentry. For deep-space missions, these options are not possible, and regular resupply is unfeasible. Controlled Ecological Life-Support Systems (CELSS) are envisioned life support systems that utilize biological systems to recycle waste and generate resources for human subsistence during long-duration space flight or in closed habitats on Earth [4]. Recent works on bioregenerative life support emphasize the feasibility of integrating microbial processes for waste remediation, nutrient recycling, and food production in a closed-loop [1,5,6,7,8,9].

Daily astronaut nutritional needs are 2300–2400 kcal with 45–65% of the intake sourced from carbohydrates, 20–35% from fats, and 1.2–1.8 g protein per kg body weight [10]. A cultivated microbial biomass has the potential to serve as a nutritional supplement to astronauts while recycling onboard waste. Many studies find single-cell protein (SCP) systems to be a viable, sustainable food source, typically producing 30–80% protein, with numerous genera at the higher end of this range [11,12,13].

Recent research has demonstrated integrated anaerobic–phototrophic membrane bioreactor systems that can treat wastewater and generate biomass [14,15]. In these systems, anaerobic microbial communities are used to effectively process solids and complex organic waste, sequester nutrients, and generate a secondary waste stream more amenable to further processing [15]. In some cases, the anaerobic reactor includes phototrophic microbial communities that increase the rate of anaerobic digestion to allow for continuous waste treatment [14]. Using an anaerobic membrane bioreactor specifically designed for space-flight applications has resulted in high carbon removal rates (95–99%) sustained over many weeks including after recovery from a simulated waste overload event [15].

**Figure 1:**
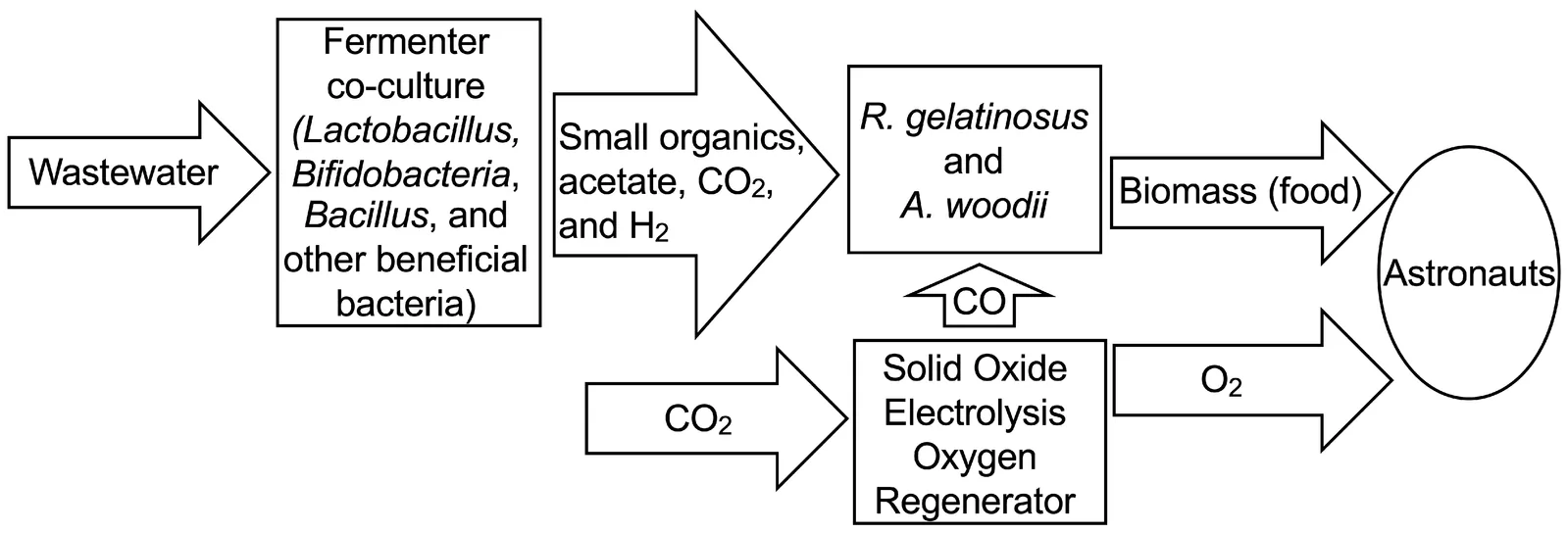
Proposed scheme for a portion of crew waste management showing the reuse of waste products and microbial biomass production.

Given the demonstrated value of anaerobic microbial processes being integrated with a phototrophic system, we here suggest a conceptual scheme to produce biomass of *Acetobacterium woodii* and *Rubrivivax gelatinosus* as a crew nutritional supplement. *R. gelatinosus* has previously been reported as a potential feedstock for tilapia [16] and has been shown to grow on various wastewaters [17,18,19] consuming acetate under anaerobic conditions [20]. The microbe has also demonstrated growth in the presence of CO [21]. The acetogen *A. woodii* has been shown to grow with CO under some conditions and can grow chemoautotrophically using CO_2_ and H_2_ [22], and various acetogens are used in initial steps of sequential fermentation processes to produce protein-rich biomass from industrial gases [23]. Because the metabolisms of both *A. woodii* and *R. gelatinosus* can produce biomass by consuming CO, these microorganisms were chosen to initially explore the proposed scheme for a portion of a CELSS during long-duration space travel. In this proposed scheme, the products of anaerobic wastewater digestion, and CO derived from CO_2_ processing are used for anaerobic growth of these two non-pathogenic anaerobic microorganisms. Previously, our group explored anaerobic digestion as a first step toward space flight waste recycling [8,24]. Due to the production of CH_4_ during typical anaerobic digestion, Steinberg et al. [8] suggested methanotrophic biomass as a nutrient supplement during long-duration space flight. However, aerobic methanotrophy consumes valuable oxygen. Therefore, as a portion of a crew waste management system, the scheme explored in this paper (Figure 1) starts with anaerobic fermentative digestion (in the absence of methanogens) producing small organic acids, H_2_, and CO_2_. Such non-methanogenic anaerobic digestion has been explored as a means of H_2_ production (e.g., [25]), but notably such digestion typically remineralizes only a portion of the total waste carbon. This paper focuses on downstream steps where products of such fermentation combined with waste CO gas (from CO_2_ processing) are converted into a potential supplemental nutritional biomass of *R. gelatinosus* and *A. woodii* cells.

Here, specifically, we investigated the plausibility of utilizing recovered nutrients and waste CO to produce *Acetobacterium woodii* and *Rubrivivax gelatinosus* biomass that could be used as a supplement for astronauts (Figure 1). First, we examined the thermodynamic feasibility of the system’s reactions. We then conducted nutritional analyses of candidate organisms. Finally, we conducted growth experiments using *R. gelatinosus* under varying acetate and ammonium concentrations. Collectively, this work contributes to the development of integrated microbial systems for nutrient recycling and food production in long-duration space flight.

## 2. Materials and Methods

### 2.1. Thermodynamic Calculations

Gibbs free energies of reaction (ΔG) were calculated in a custom Python (2.7.14) script based on Standard Gibbs Free Energies of formation and varying environmental conditions. The Standard Gibbs Free Energy of reaction (ΔG°) was calculated according to the equation:

ΔG° = ΔG°_products_ − ΔG°_reactants_ (1)

where all constituents are at activities = 1, pressure = 1 bar, and temperature = 298 K. Deviations from the Standard Gibbs Free Energy are given by the equation:

ΔG = ΔG° + RT ln(Q)(2)

where R is the ideal gas constant, T is the temperature in Kelvin, and Q is the reaction quotient, defined as the product of activities of products raised to their molar coefficients divided by the product of activities of reactants raised to their molar coefficients. Henry’s Law is used to determine the concentration of gases dissolved in water at a given pressure and temperature. ΔG is calculated over varying temperatures, pressures, and concentrations of reactants/products. Standard Gibbs Free Energy (ΔG°) was converted to Biochemical Standard Gibbs Free Energy (ΔG°′) according to the equation:

ΔG°’ = ΔG° + *m*RT ln(10^−7^) (3)

where *m* is the number of protons involved in the reaction (negative for more protons consumed than formed) [26]. The thermodynamic results shown in output graphs explore ranges of parameters that may allow for favorable forward reactions in a theoretical bioreactor.

### 2.2. Nutritional Analyses

*A. woodii* WB1 (DSM 1030) and *R. gelatinosus* NCIB 8290 (DSM 1709) were purchased from The Leibniz Institute DSMZ-German Collection of Microorganisms and Cell Cultures (https://www.dsmz.de/) (accessed on 20 July 2026) and grown under anaerobic conditions. *A. woodii* was grown in 50 mL of DSMZ medium 135 with the fructose and resazurin omitted and with a 200 kPa headspace of 80% H_2_ and 20% CO_2_. *R. gelatinosus* was grown in 50 mL of DSMZ 27 medium with the resazurin and succinate omitted (as described in the next section as the “Low acetate” condition). The analyses of these two type strains were performed in triplicate using three independent growth bottles for each microorganism using a 0.5 mL inoculation of a pre-culture grown to the late-log growth phase.

For the percent moisture, a cell pellet was resuspended and washed with pH 7.2 phosphate-buffered saline (PBS) and centrifuged before the wet mass was measured. Then, the pellet was dehydrated (110 °C for over 24 h) and the dry mass measured. To determine the percent lipid, a washed cell pellet was lyophilized and twice extracted with chloroform/methanol/lipid-free water in a 1:2:0.8 *v/v* ratio. After separating overnight, the lipid fraction was collected and dried. The washed cell pellets were lysed using a buffer of 10 mM tris-hydrochloride (Tris-HCl), 0.1% Triton X-100, 0.2 mg/mL lysozyme, and 1x protease inhibitor solution. The percent protein measurement of lysed *A. woodii* was found using a Lowry protein assay kit. Due to pigmentation interference, the Direct Detect Spectrometer (MilliporeSigma, Burlington, Massachusetts) infrared protein quantitation system was used for *R. gelatinosus* with the lysed cell sample being diluted with PBS so the measured protein concentration fell into the instrument’s range. Direct Detect measurement was performed by the Penn State Automated Biological Calorimetry Facility.

### 2.3. Primary Growth Experiment–Varying Substrate Levels

For *R. gelatinosus* NCIB 8290 (DSM 1709), DSMZ 27: “Rhodospirillaceae Medium (modified) media” was used with resazurin and succinate omitted, yeast extract reduced to 0.03 g/L, and acetate reagents varied as described below. In all cases, the solution included the regular 0.4 g/L ammonium chloride. The media pH was adjusted to 6.8, sparged with N_2_ gas, and anaerobically distributed to butyl-stoppered bottles (50 mL of media per bottle). Bottles were filled with an inert 1.5 bar N_2_ headspace, and autoclaved. To mimic growth under conditions where acetate and ammonium concentrations are correlated (such as wastewater), three media with different levels of acetate reagents were made: no ammonium acetate added (0 mM total acetate and 7.5 mM total ammonium), “low ammonium acetate” with 0.50 g ammonium acetate and 1.00 g sodium acetate per liter (18.7 mM total acetate and 14.0 mM total ammonium), and “high ammonium acetate” with 1.00 g ammonium acetate and 2.00 g sodium acetate per liter (37.4 mM total acetate and 20.5 mM total ammonium). Experiments were inoculated with 0.5 mL of a pre-culture grown to the late-log growth phase. One “dark” control for each type of medium was prepared and incubated under the same conditions but completely wrapped in aluminum foil. In addition to the three bottles of each condition incubated in the light and one dark bottle per condition, one uninoculated control for each medium was also prepared and incubated. Inoculated bottles were incubated in light at 30–32 °C with 120 rpm shaking. Incubation was done in an Innova 4340 Illuminated Incubator shaker (New Brunswick Scientific, Edison, New Jersey) with full illumination (in this case, the flux was 1200 Lux).

Aliquots of the bottles were taken for analysis by optical density (OD) measurements with a UV-vis spectrophotometer set to a wavelength of 600 nm and for cell counts using phase contrast microscopy and a hemocytometer.

Before and after growth, acetate concentrations were determined for *R. gelatinosus* filtered samples via ion chromatography (by the external laboratory Alfa Chemistry).

### 2.4. Second Growth Experiment–Variable Acetate vs. Ammonium

With 50 mL per bottle, *R. gelatinosus* NCIB 8290 (DSM 1709) was grown under four different conditions to illuminate the separate growth effects of acetate and ammonium concentrations. There was a “low acetate, low ammonium” condition that was identical to the “low acetate” growth condition in the initial study (18.7 mM total acetate and 14.0 mM total ammonium). There was a “high acetate, high ammonium” condition that was identical to the “high acetate” growth condition in the initial study (37.4 mM total acetate and 20.5 mM total ammonium). There was a “high acetate, low ammonium” growth condition with a total of 2.53 g/L sodium acetate used (37.4 mM total acetate and 14.0 mM total ammonium). Lastly, there was a “low acetate, high ammonium” condition (18.7 mM total acetate and 20.5 mM total ammonium) where ammonium chloride totaled 0.75 g/L, but other reagents were left identical to the “low acetate” growth condition. There was one uninoculated blank and three inoculated replicates grown for each medium type. Experiments were inoculated with 0.5 mL of a pre-culture grown to the mid-log growth phase. Incubation matched the temperature and shaking settings of the initial study. Absorbance at 600 nm was used to monitor growth throughout the experiment. Cell counts were taken on the final day of the study.

## 3. Results

### 3.1. Proposed Scheme and Thermodynamic Calculations

The first reaction of our proposed scheme (Table 1) produces CO from CO_2_ according to Rxn. 1:

2CO_2_ → 2CO + O_2_ (Rxn. 1)

The generation of O_2_ from CO_2_ (to resupply O_2_ recycled from crew respired CO_2_) using solid oxide electrolysis is a process that co-produces CO as a byproduct [27,28,29,30,31]. This reaction has a Standard Gibbs Free Energy of 257 kJ/mol CO_2_. With the investment of energy already required to produce crew O_2_, additional energy is not needed to use the CO byproduct (waste) gas as feeding gas for microbial bioreactors.

Wastewater typically contains a variety of carbohydrates, lipids, and proteins, but here Rxn. 2 uses glucose fermentation to produce acetate as a model illustrating non-methanogenic anaerobic waste digestion:

C_6_H_12_O_6_ + 4H_2_O → 2C_2_H_3_O_2_^−^ + 2HCO_3_^−^ + 4H_2_ + 4H^+^ (Rxn. 2)

Such microbial fermentations produce a variety of organic acids, H_2_, and CO_2_ [32,33]. The resultant products of digestion are proposed to be used to feed other reactions defined below.

The anaerobic bacterium *A. woodii* forms acetate from a variety of substrates, including a CO headspace or a gas mixture of H_2_ and CO_2_ [34,35]. The waste CO produced by Rxn. 1 is used to feed a carboxydotrophic acetogenic metabolism (Rxn. 3) to produce microbial biomass (e.g., *A. woodii*):

4CO + 4H_2_O → C_2_H_3_O_2_^−^ + 2HCO_3_^−^ + 3H^+^ (Rxn. 3)

**Figure 2:**
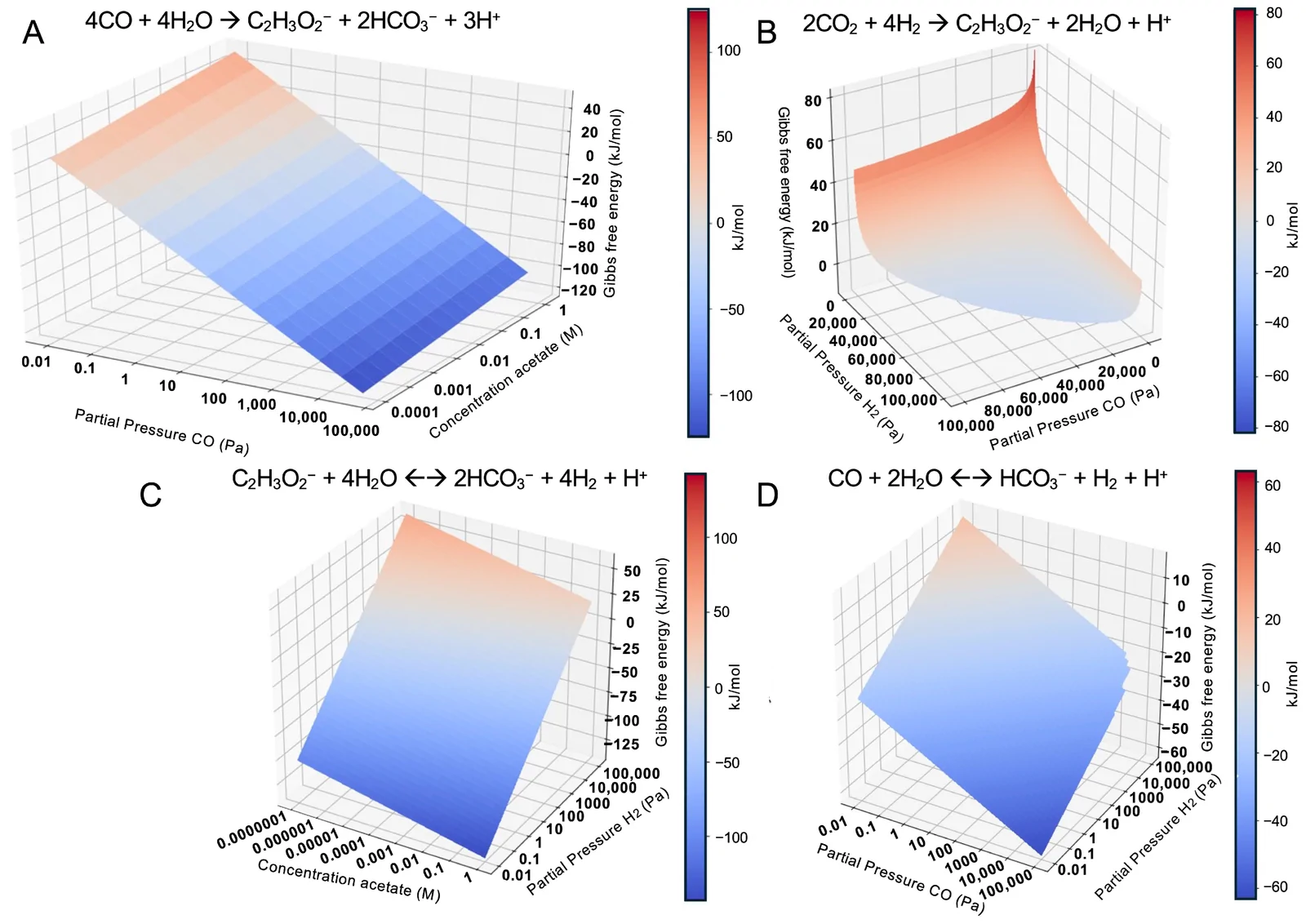
Gibbs free energy (ΔG) of relevant reactions. [HCO_3_^−^] = 0.1 M, pH = 7, T = 30 °C, H_2_O activity = 1. Total P = 10^5^ Pa with partial pressures assumed to be balanced by inert gas. (**A**) ΔG for Rxn. 3 producesacetate from CO. Bicarbonate constant at 0.1 M, pH = 7, 30 °C. (**B**) ΔG for Rxn. 4 producing acetate from CO_2_ and H_2_. Water activity = 1, [acetate] = 10^−3^ M, pH = 7, T = 30 °C. (**C**) ΔG for Rxn. 5 showing the consumption of acetate producing H_2_. Water activity = 1, [HCO_3_^−^] = 0.1 M, pH = 7, T = 30 °C. (**D**) ΔG for the net reaction for the production and consumption of acetate (combination of Rxns. 3 and 5).

Figure 2A shows the ΔG for Rxn. 3 where CO converts to acetate. We find that the reaction is favorable over a wide variety of conditions (headspace CO > ~1 Pa and pH ~7 at 30 °C).

Biomass (e.g., *A. woodii*) and acetate are also formed by hydrogenotrophic acetogenic reduction of CO_2_ (Rxn. 4):

2CO_2_ + 4H_2_ → C_2_H_3_O_2_^−^ + 2H_2_O + H^+^ (Rxn. 4)

Figure 2B explores the thermodynamics of this microbial reaction using a range of partial pressures of both reactant gases at 30 °C and a pH of 7. We find that the headspace partial pressure of H_2_ can be as low as ~1.5 × 10^4^ Pa with at least ~2500 Pa headspace pressure of CO_2_.

The next microbial reaction, which is unfavorable at standard conditions, consumes acetate and produces H_2_ (Rxn. 5) and is performed by *R. gelatinosus* grown photoheterotrophically:

C_2_H_3_O_2_^−^ + 4H_2_O → 2HCO_3_^−^ + 4H_2_ + H^+^ (Rxn. 5)

Purple non-sulfur bacteria, such as *R. gelatinosus*, can grow photoheterotrophically under anaerobic conditions, utilize organic acids such as acetate, and produce biomass, CO_2_, and H_2_ [36,37,38,39]. At pH 7 and 30 °C, the reaction is favorable (Figure 2C) for consuming acetate for a wide range of H_2_ partial pressures (if the pH_2_ is less than ~10^4^ Pa).

An example net reaction for microbial acetate production and consumption (Table 1) was explored in Figure 2D. By producing acetate from CO and consuming it to form H_2_, the acetate on either side of the equation cancels out leaving the conversion of CO and water to form bicarbonate, H_2_, and H^+^. The reaction is favorable over several orders of magnitude of CO and H_2_ partial pressures (requiring at least ~1 Pa of CO to be available).

### 3.2. Nutritional Analyses

Nutritional contents for both *R. gelatinosus* and *A. woodii* were determined (Table 2). *A. woodii* contained a greater percentage of moisture compared to *R. gelatinosus*, but a lower protein and lipid content. *R. gelatinosus* biomass consisted of 54% protein and 21% lipid, as a dry mass. *A. woodii* biomass consisted of 21% protein and 11% lipid on a dry mass basis.

### 3.3. Primary Growth Experiment–Varying Substrate Levels

**Figure 3:**
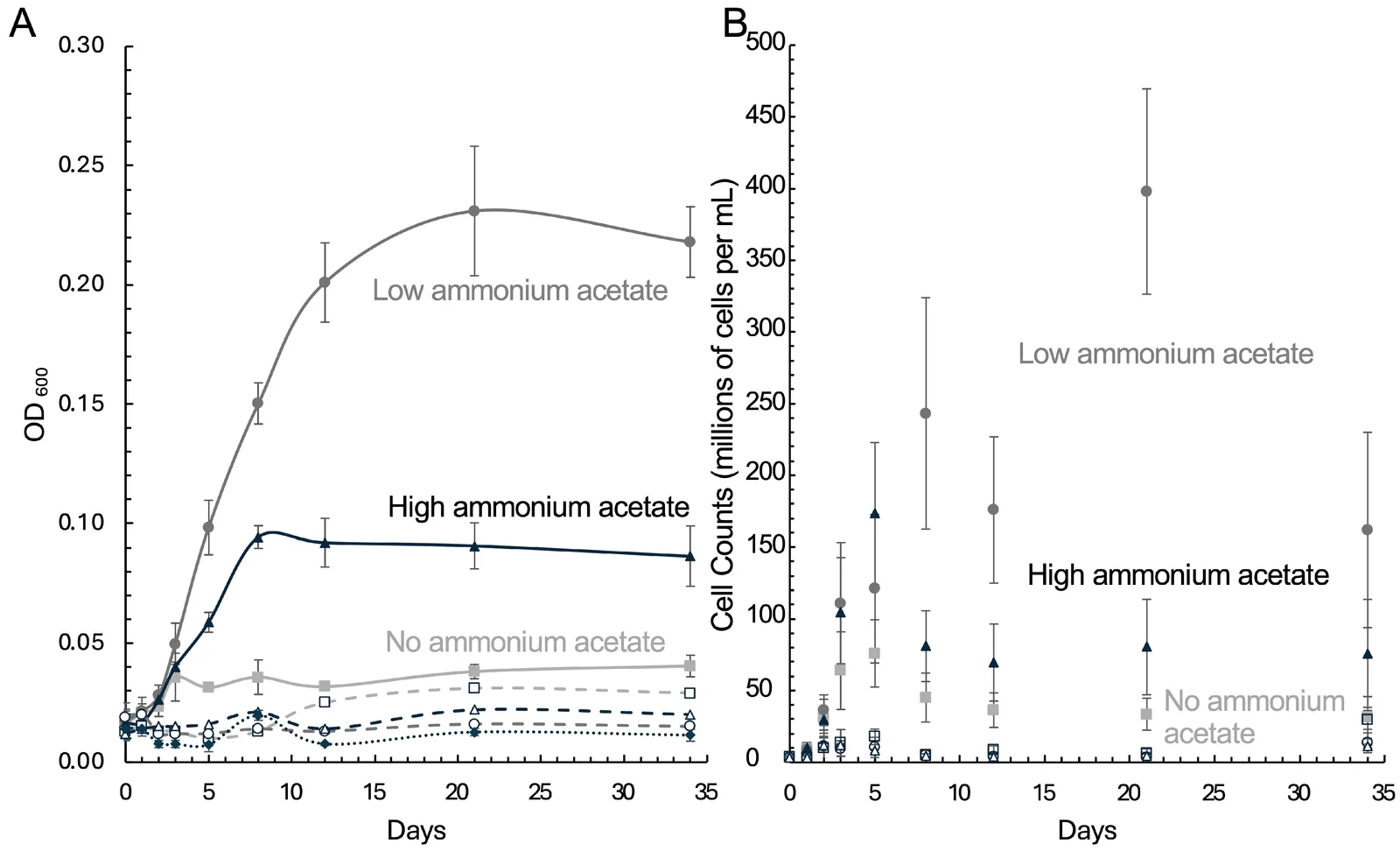
Growth of *R. gelatinosus* provided “No ammonium acetate” (0 mM total acetate and 7.5 mM total ammonium; squares), “Low ammonium acetate” (18.7 mM total acetate and 14.0 mM total ammonium; diamonds), and “High ammonium acetate” (37.4 mM total acetate and 20.5 mM total ammonium; triangles) with light (solid symbols and solid lines) and without light (open symbols and dashed lines). Each data point represents the average ±1 SD of measurements on three replicate growth bottles. The average ± 1 SD of the controls (dotted line), which consisted of an uninoculated bottle for each light experiment, is shown. (**A**) Optical density (OD_600_) as a function of incubation time in days. The data are connected with smoothed lines to provide clarity. (**B**) Direct cell counts (millions of cells per mL) as a function of incubation time in days. Controls not shown as they had no observable cells.

Initial *R. gelatinosus* growth experiments using media containing different levels of ammonium acetate demonstrate heterotrophic photosynthetic growth based on acetate as a carbon source. Figure 3A indicates there was no growth in uninoculated blanks or in the inoculated bottles incubated in “dark” conditions. Minor growth was seen in the no ammonium acetate added condition, moderate growth in the “high” ammonium acetate condition, and the highest growth was seen in the “low” ammonium acetate condition. Cell count data (Figure 3B) from the same experiments are in agreement with the findings based on absorbance data. Acetate concentrations of filtered *R. gelatinosus* media were measured before and after growth (Table 3).

### 3.4. Second Growth Experiment–Variable Acetate vs. Ammonium

**Figure 4:**
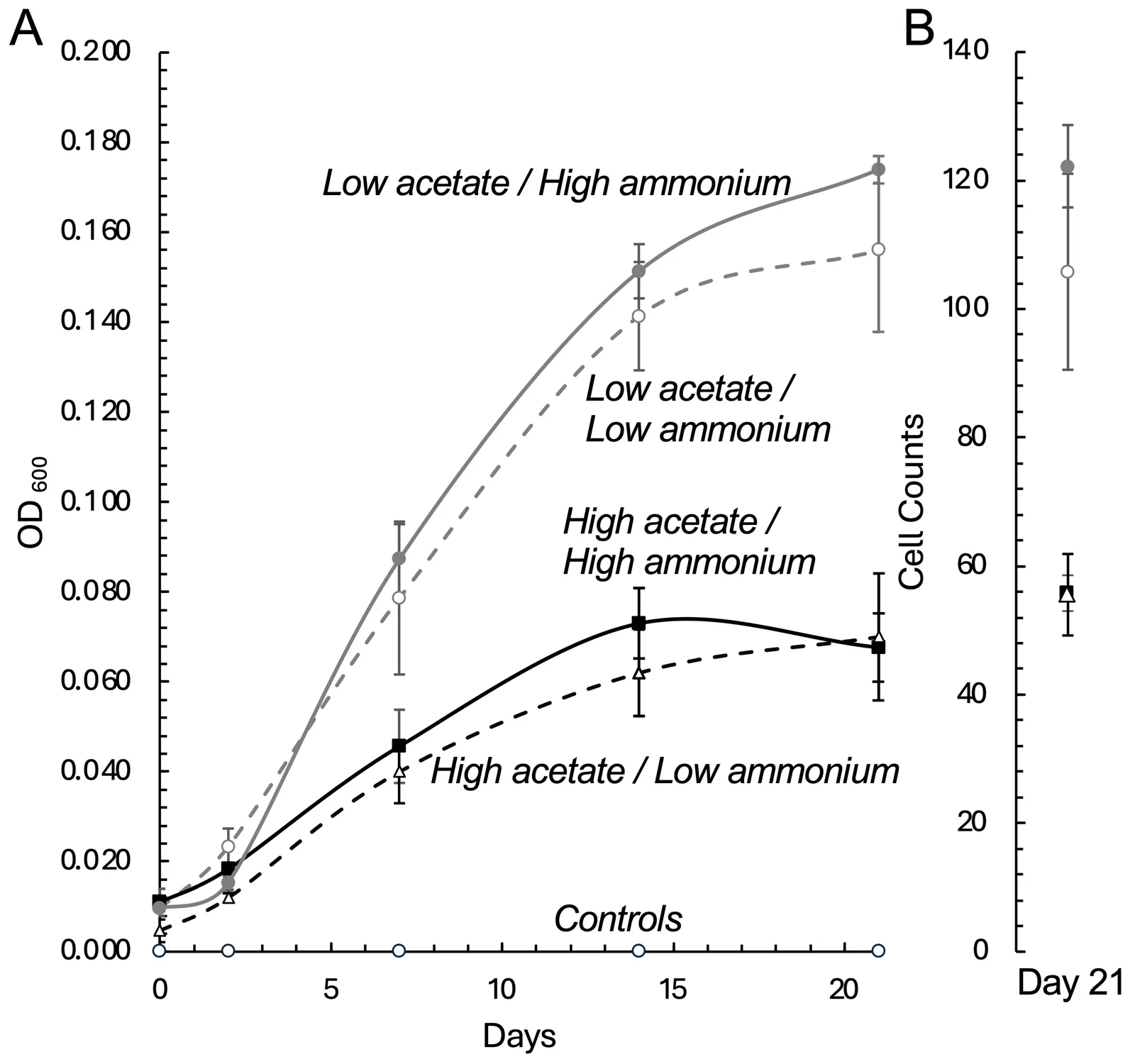
Growth of *R. gelatinosus* incubated in light with separately varied acetate (18.7–37.4 mM) and varied ammonium concentrations (14.0–20.5 mM) including “Low acetate/Low ammonium” (gray open circles and dashed line), “Low acetate/High ammonium” (gray circles and solid line), “High acetate/Low ammonium” (black open triangles and dashed line), and “High acetate/High ammonium” (black squares and solid line). (**A**) OD_600_ over three weeks. Each data point represents the average ±1 SD of measurements from three replicate growth bottles. (**B**) Direct cell counts (millions of cells per mL) from the final day of the secondary growth experiment (day 21). Controls are not shown as they had no observable cells.

The second experiment compared the effect of acetate versus ammonium on the growth of *R. gelatinosus.* Figure 4A shows optical density results from inoculated bottles. Cell counts from Day 21 are shown in Figure 4B, which supports results of absorbance data.

## 4. Discussion

### 4.1. Proposed Scheme and Thermodynamic Calculations

Overall, the proposed scheme uses CO derived from CO_2_ processing and microbial metabolisms to produce biomass of *A. woodii* and *R. gelatinosus* as crew nutritional supplements. In the proposed scheme for crew life support, crew O_2_ would be produced through solid oxide electrolysis. While this process is thermodynamically unfavorable, it should be reliable given sufficient energy availability. In fact, Rxn. 1 has been tested on Mars [30,31] for in situ resource utilization where the predominantly CO_2_ atmosphere can be used to produce O_2_ [27] with CO as a byproduct. The scheme suggested here uses wastewater digestion by fermenters to process crew waste. Shown as Rxn. 2, this process is thermodynamically favorable under anaerobic conditions resulting in potential feedstock for further microbial activity, including the growth of non-pathogenic microbial biomass.

For potential food production, the thermodynamics of Rxn. 3 indicate that the acetogen *A. woodii* should be able to grow, producing biomass and acetate whenever CO is available from O_2_ production. Previous work has indicated that acetogens, such as *A. woodii*, can grow on CO, although a co-substrate, such as formate, may be required [22]. Providing such required co-substrates is plausible, given that formate is formed during wastewater digestion. Acetogens can also grow using Rxn. 4, which we found to have minimum H_2_ pressure requirements that were significantly higher than in the CO-driven metabolism with the assumptions made in our calculations, although experimental work indicates that A. woodii can grow at pH_2_ as low as 250 Pa [40]. The results suggest H_2_ may need to be collected and concentrated from our other proposed processes (including both Rxn. 2 waste digestion and Rxn. 5 *R. gelatinosus* growth) to feed to *A. woodii* for a self-contained system producing hydrogenotrophic biomass. In Rxn. 5, photoheterotrophic growth of *R. gelatinosus* produces microbial biomass as a crew nutritional supplement. Purple non-sulfur bacteria, such as *R. gelatinosus*, are of particular interest as sources for single-cell protein due to their metabolic versatility. While we find this reaction favorable under a range of low H_2_ conditions, it is likely that this reaction only proceeds microbially in the presence of light considering that *R. gelatinosus* uses light to drive the photoheterotrophic reaction shown. Because *R. gelatinosus* can tolerate and oxidize CO [21,41,42], this microbial species would likely be able to survive in a proposed, but untested, co-culture with *A. woodii* that is being fed CO and producing acetate, as shown in Figure 1. Feeding H_2_ from Rxn. 5 to the reaction of Rxn. 3 in such a co-culture could hypothetically result in a photo-dependent loop sustainable until a large portion of the available carbon and hydrogen is contained in biomass.

Overall, our thermodynamic modeling demonstrated that key reactions linking acetate, CO, and H_2_ metabolisms can proceed under conditions relevant to bioreactor operation, suggesting that the proposed metabolic network is energetically viable. Similarly, systems-level analyses of closed microbial ecological life support systems emphasize the importance of coupling multiple metabolic processes to achieve resource utilization [43].

### 4.2. Nutritional Analyses

Purple non-sulfur bacteria have been shown to generate protein-rich biomass [44,45,46,47,48], and *R. gelatinosus*, in particular, has been studied as a supplemental nutritional feed for fish and poultry [16,17,18,48]. We found that *A. woodii* contained a greater percentage of moisture compared to *R. gelatinosus*, but a lower protein and lipid content. The nutritional analysis of *R. gelatinosus* and *A. woodii* highlights the potential of these organisms as sources of single-cell protein, with *R. gelatinosus* exhibiting particularly high protein and lipid content. The values we found for each bacterial species are consistent with broader studies of microbial protein systems [12,49].

Alternatively, instead of being a direct food ingredient or nutritional supplement, the biomass could be a feed for aquaculture or an agricultural soil amendment [50,51] during space flight. Safety considerations, however, remain critical for the deployment of microbial biomass as food in space. In particular, the nucleic acid content of microbial biomass and potential accumulation of purines must be managed to prevent adverse health effects [49]. Primary concerns regarding SCP in general are high uric acid content, although high purine diets might not cause gout without underlying disease, particularly from non-animal sources [52].

### 4.3. Primary Growth Experiment–Varying Substrate Levels

Our primary growth experiment using *R. gelatinosus* showed photoheterotrophic growth utilizing acetate as the microbe’s carbon source. Surprisingly, the cultures with a moderate amount of added ammonium acetate grew notably better than the cultures with a higher level of added ammonium acetate (at 21 days, *t*-test *p* = 0.0011 for OD_600_; *t*-test *p* = 0.0022 for cell counts). The measurements of acetate before and after growth show similar acetate consumption by *R. gelatinosus* in “high ammonium acetate” and “low ammonium acetate” bottles (difference = 24 ± 213; *t*-test *p* = 0.86; e.g., not significantly different). These results suggest that the “high ammonium acetate” cultures were using a similar, if not higher, amount of substrate even though they produced notably less biomass in the process than the cells thriving in the “low ammonium acetate” condition. In these experiments, the “dark” conditions did not show meaningful growth, supporting *R. gelatinosus’s* reliance on photosynthesis. There was slight growth in the no ammonium acetate condition under illumination, which was most likely due to the minimal yeast extract included in all media types.

Overall, this experiment suggested that acetate concentration plays a critical role in biomass production. Moderate concentrations support optimal growth and higher concentrations led to inhibited growth.

### 4.4. Second Growth Experiment–Variable Acetate vs. Ammonium

Following up on the results of the primary growth experiment, the second experiment compared the effects of acetate versus ammonium on the growth of *R. gelatinosus* using four different media conditions in which the acetate and ammonium concentrations were varied independently. The two media types with “low acetate” had more robust growth (OD_600_ > 0.14 and >10^8^ cells/mL at 21 days) than both of the “high acetate” cultures (OD_600_ < 0.08 and <0.6 × 10^8^ cells/mL at 21 days). For example, at 21 days, the OD_600_ growth of “Low acetate/Low ammonium” was higher than that of “High acetate/High ammonium” with *t*-test *p* = 0.0015. Cell counts from Day 21 are shown in Figure 4B, which supports the results of the absorbance data. For the same example comparison of growth, the cell count *t*-test *p* = 0.0055.

Taken as a whole, this second growth experiment found that the “high acetate” condition had an inhibitory effect on the growth of *R. gelatinosus*, but with this preliminary data, the cause of this difference in growth is uncertain. The result is surprising considering acetate is the main organic carbon source available in the media. Yet, these results can be explained by the relatively low need for carbon compared to energy. Similar substrate inhibition effects are widely reported in single-cell protein systems and are often governed by interactions among carbon availability, osmotic stress, and metabolic regulation rather than simple toxicity [13,16].

## 5. Conclusions

To address weight and space restrictions for long-term space missions, new approaches and technologies such as single-cell food sources that can be produced in situ from available waste products will be needed. We suggest here in this perspective that a recycled nutrient model that complements solid oxide electrolysis is a reasonable possibility. The microbes studied, *A. woodii* and *R. gelatinosus*, are plausible food sources based on our preliminary nutritional analyses. Of these, *R. gelatinosus* is particularly interesting due to its high protein content of 54%. Experiments show photosynthetic growth of *R. gelatinosus* is dependent on the presence of acetate when other organics are not provided. Growth inhibition at high levels of acetate, however, highlights the challenges of microbial bioreactors for producing crew food and the need for further research. This study should encourage further investigation of photoheterotrophic co-cultures grown on waste byproducts as a source of single-cell protein for direct dietary supplementation or as part of an amendment to an agricultural or aquaculture system for space flight.

## Figures and Tables

**Table 1:** Relevant reactions to feed microbes from available resources in scheme [26]. Biochemical Standard Gibbs Free Energies (ΔG°’) of reactions are presented in kJ/mol of reaction at pH = 7.

Rxn. #	Reaction	ΔG°’ (kJ/mol)	Key Reaction Products	Process/ Microbe
1	2CO_2_ → 2CO + O_2_	514	CO	Solid Oxide Electrolysis
2	C_6_H_12_O_6_ + 4H_2_O → 2C_2_H_3_O_2_^−^ + 2HCO_3_^−^ + 4H_2_ + 4H^+^	−206	Organic acids & H_2_	Various
3	4CO + 4H_2_O → C_2_H_3_O_2_^−^ + 2HCO_3_^−^ + 3H^+^	−166	Acetate	*A. woodii*
4	2CO_2_ + 4H_2_ → C_2_H_3_O_2_^−^ + 2H_2_O + H^+^	−95	Acetate	*A. woodii*
5	C_2_H_3_O_2_^−^ + 4H_2_O → 2HCO_3_^−^ + 4H_2_ + H^+^	104	H_2_	*R. gelatinosus* (photo-heterotrophy)
Net of 3 & 5	CO + 2H_2_O → HCO_3_^−^ + H_2_ + H^+^	−15.3		*A. woodii &* *R. gelatinosus*

**Table 2:** Nutritional data for *A. woodii* and *R. gelatinosus.*

	% Moisture	% Protein	% Lipid
*A. woodii*	96.17 ± 0.49	21.35 ± 2.74	11.49 ± 1.81
*R. gelatinosus*	88.59 ± 0.68	54.12 ± 3.88	21.38 ± 4.12

**Table 3:** Acetate content of filtered *R. gelatinosus* media (average ± SD of 3 replicate growth bottles). Not detected (ND) indicates concentrations below 100 ppb.

Media	Acetate Before Growth (ppm)	Acetate After Incubation (ppm)
No acetate	2.6 ± 0.7	ND
Low acetate	812 ± 89	732 ± 75
High acetate	1544 ± 29	1440 ± 176
Control, low acetate	752	776
Control, high acetate	1404	1491

## Data Availability

The data shown in the figures of this paper are available at the Penn State data Commons. https://doi.org/10.26208/zrza-ta96 (accessed on 20 July 2026).

## Abbreviations

The following abbreviations are used in this manuscript:

ISS	International Space Station
SCP	Single-cell protein
PBS	Phosphate-buffered saline
OD	Optical density
